# Antiviral efficacy of entecavir for hepatitis B virus rtA181V/T mutants

**DOI:** 10.1186/s12985-017-0739-z

**Published:** 2017-04-04

**Authors:** Ping Li, Jiabao Geng, Wei Li, Xiaobing Xu, Xin Zhang, Wenkai Zheng, Yuecheng Yu, Zhiguo Yang, Maorong Wang

**Affiliations:** 1 Department of Liver Disease Center, Bayi Hospital Affiliated Nanjing University of Chinese Medicine, Nanjing, Jiangsu Province China; 2 Department of Infection Disease Center, Henan Province People’s Hospital, Zhengzhou, Henan Province China; 3 Department of Gastroenterology and Hepatology, Nanjing General Hospital of Nanjing Military Command, Nanjing, Jiangsu Province China

**Keywords:** Chronic hepatitis B, Nucleoside and nucleotide analogs (NAs), Entecavir, Mutation

## Abstract

**Background:**

The amino acid substitution at position 181 of the Hepatitis B virus (HBV) polymerase is a multi-drug resistance affecting both the L-nucleoside and acyclic phosphonate nucleotide groups. Data is limited on the efficacy of entecavir (ETV) rescuing chronic hepatitis B (CHB) patients with rtA181T/V mutation.

**Methods:**

Thirty-one patients with rtA181T/V mutation and 25 patients with rtA181T/V and rtN236T mutation were enrolled. Virological, serological and biochemical outcomes of ETV rescue therapy over 12 months in CHB patients with rtA181T/V mutation strains were investigated. All patients were treated with ETV 0.5 mg/day for 12 months and scheduled follow-up every 3 months. Patients’ characteristics, laboratory tests results and clinical outcomes were collected and compared.

**Results:**

After emergence of rtA181T/V mutant, serum HBV DNA levels increased over 4 log10 IU/mL, but the total bilirubin, alanine aminotransferase (ALT) levels raised moderately. No significant difference in baseline characteristics was observed between the rtA181T/V group and rtA181T/V + rtN236T group. After 12 months rescue therapy, total 85.7% (48/56) patients achieved HBV DNA undetectable. No significant difference in the mean reduction of serum HBV DNA and biochemical response was observed between both groups (3.59 ± 1.85 vs. 3.76 ± 2.15 log10 IU/ml; *P* = 0.756 and 90.3 vs. 80.0%; *P* = 0.272, respectively). The mean HBV DNA reduction, HBsAg and ALT levels were also similar between different rtA181T/sW172 mutations (*P* > 0.05). HBV DNA level is the only predictor of 12 months antiviral outcomes (odds ratio 6.723, *P* = 0.022).

**Conclusions:**

The results of the present study suggested that ETV is efficient in rescuing rtA181T/V mutation CHB patients. HBV DNA level could predict viral clearance at the 12^th^ month.

## Background

Hepatitis B virus (HBV) infection is a major health problem that caused over 350 million people suffering chronic infection worldwide [1, 2]. Nucleos(t)ide analogs (NAs) including lamivudine (LAM), adefovir dipivoxil (ADV), entecavir (ETV), telbivudine (LDT) and tenofovir(TDF) are currently approved to suppress HBV replication so as to prevent the progression of liver diseases. However, NAs need sustained treatment because it can not remove covalently closed circular DNA in the nucleus. During the long-term antiviral treatment, drug resistance begins with mutations in the polymerase gene, leading to an increase in viral load and serum alanine aminotransferase (ALT) levels, progression of liver disease, even causing liver cirrhosis or failure. Drug resistance has become a major problem during antiviral treatment.

According to resistance patterns, NAs drugs can be structurally grouped as L-nucleoside (LAM, LDT), acyclic nucleotide phosphonate (ADV, TDF), and D-cyclopentanes (ETV). The amino acid substitution at position 181 of the HBV polymerase is a multi-drug resistance which could affect both the L-nucleoside and acyclic phosphonate nucleotide groups [3]. Additionally, *in vitro* studies have found the rtA181T/V mutation could affect virus replication [4], protein secretion [5] and tumorigenesis [6, 7]. Switching to ETV for rtA181T/V mutation patients was recommended by the Asian Pacific Association for the Study of the Liver (APASL) [8]. But few studies reported the effect of ETV in rescuing rtA181T/V mutation patients.

In general, the majority of HBV rtA181V/T mutants are known to be induced after ADV therapy, along with the rtN236T mutant. Therefore, we aimed to evaluate the antiviral efficacy of 12 months ETV rescue therapy in rtA181T/V single mutation or with rtN236T mutant.

## Methods

### Patients

A total number of 56 CHB patients, who visited our hospital during January 2010 to May 2014, were enrolled in this study. They developed rtA181T/V mutation strains during ADV treatment. Among them, 31 patients developed rtA181T/V mutation strains (rtA181 group), and the other 25 patients developed rtA181T/V and rtN236T mutation strains (rtA181 + rtN236 group). No other HBV RT mutants were detected except rtA181 and rtN236. These patients were treated with 0.5 mg/day ETV for at least 12 months and scheduled follow-up every 3 months. Patients who received IFN-α treatment or co-infected with hepatitis C, hepatitis D, or human immunodeficiency virus were excluded from this study. The diagnostic criteria were based on the Management Scheme of Diagnostic and Therapy Criteria of Viral Hepatitis issued by the Chinese Society of Infectious Diseases. This study was approved by the institutional review board of China Nanjing bayi hospital and all aspects of the study comply with the Declaration of Helsinki. The Ethics Committee specifically approved that no informed consent was required because this was a retrospective study and the data were analyzed anonymously.

### Serum assay

ALT, aspartate aminotransferase (AST), and total bilirubin in serum were measured by the Olympus AU5400 Chemistry System (Beckman Coulter, Switzerland). Viral markers, including hepatitis B surface antigen, hepatitis B e antigen (HBeAg), and anti-HBe, were tested by Chemiluminescent Assay (Architect i2000SR; Abbott Diagnostics, Abbott Park, IL, USA). HBV DNA was quantified by real-time PCR using a commercial detection kit (Kehua Bio-Engineering) with the aid of Light Cycler Detection System (ABI 7300 Realtime PCR system, USA). The lower limit of detection for this assay is 50 IU/mL.

HBV resistance mutations were identified by DNA sequencing. The fragments (nt130–nt1129), encompassing the reverse transcriptase (RT) domain, were amplified by nested PCR. The first pair of primers for the PCR were 5′-AAGCTCTGCTAGATCCCAGAGT-3′ (sense: nt18–nt40) and 5′-TTTCGCTCCAGACCGGCTGC-3′ (antisense: nt1320–nt1301). The second pair of primers for the PCR included 5′-GCGGGGTTTTTCTTGTTGAC-3′ (sense: nt56–nt75) and 5′-AGTATGGATCGGCAGAGGAG-3′ (antisense: nt1272–nt1253). PCR products were directly sequenced on an automated DNA sequencer (ABI 3730XL, USA) using the second sense primer. Substitutions at positions rt80, rt173, rt180, rt181, rt184, rt202, rt204, rt236, and rt250 were taken as resistance mutations for analysis [9].

### Statistical analysis

Serum HBV DNA levels and HBsAg titers were expressed on a logarithmic scale. Quantitative data were expressed as means ± standard deviations and analyzed by independent samples *t* test or the Mann–Whitney *U* test. Count data constitution were analyzed by chisquared test. Multivariate analysis was performed by a logistic regression model. Statistical analysis was carried out with the aid of SPSS 13.0 software. The significance was set at *P* < 0.05.

## Results

### Study population

In 56 CHB patients appeared rtA181T/V mutation, 51 (91.1%) were male and 5 (8.9%) were female, and the mean age was 42.1 ± 8.6 years. After emergence of rtA181T/V mutant, mean serum HBV DNA increased over 4 log_10_ IU/mL. There were no statistically significant differences between these two groups regarding mean age, sex ratio, liver functions, HBV DNA level, HBsAg titer and HBeAg positive rate. The baseline characteristics of the two study groups were presented in Table 1.

**Table 1:** Characteristics of HBV rtA181T/V mutant patients

Characteristics	rtA181 group (*n* = 31)	rtA181 + rtN236 group (*n* = 25)	*P* value
Age (years)			
Mean ± SD	42.4 ± 9.5	41.7 ± 7.6	0.763
Sex (No.)			
Male/Female	27/4	24/1	0.245
Total bilirubin level (μmol/L)			
Mean ± SD	17.2 ± 8.0	16.2 ± 8.2	0.671
ALT (U/L)			
Mean ± SD	81.4 ± 88.4	162.2 ± 316.4	0.226
AST (U/L)			
Mean ± SD	47.0 ± 31.4	79.8 ± 124.7	0.210
HBV DNA (log_10_ IU/mL)			
Mean ± SD	4.15 ± 1.87	4.35 ± 1.71	0.678
HBsAg (log_10_ IU/ml)			
Mean ± SD	3.57 ± 0.37	3.63 ± 0.39	0.561
HBeAg (No.)			
Positive/Negative	24/7	18/7	0.642

### Virological responses

The mean changes in serum HBV DNA at 6th month and 12th month were shown in Table 2. According to baseline, the mean serum HBV DNA at 6^th^ month and 12^th^ month were significantly reduced not only in rtA181 group but also in rtA181 + rtN236 group (*P* < 0.001). However, the mean reduction of serum HBV DNA at 6^th^ month did not differ significantly between the rtA181T/V group (3.18 ± 1.92 log10 IU/ml) and the rtA181 + rtN236 group (3.52 ± 2.04 log10 IU/ml) (*P* = 0.520). In addition, no statistical difference in the mean decline of serum HBV DNA at 12^th^ month was observed between the two groups (3.59 ± 1.85 vs. 3.76 ± 2.15 log10 IU/ml; *P* = 0.756).

**Table 2:** Virological and Biochemical Responses During ETV Rescue Therapy

Response	rtA181 group (*n* = 31)	rtA181 + rtN236 group (*n* = 25)	*P* value
Virological			
HBV DNA Reduction (log_10_ IU/mL)			
6^th^ Month (Mean ± SD)	3.18 ± 1.92	3.52 ± 2.04	0.520
12^th^ Month (Mean ± SD)	3.59 ± 1.85	3.76 ± 2.15	0.756
undectectable			
6^th^ Month (No. %)	24/31 (77.4%)	19/25 (76.0%)	0.900
12^th^ Month (No. %)	27/31 (87.1%)	22/25 (88.0%)	0.919
HBeAg loss			
6^th^ Month (No. %)	3/24 (1.3%)	1/18 (5.6%)	0.448
12^th^ Month (No. %)	5/24 (20.8%)	2/18 (11.1%)	0.403
Biochemical			
Total bilirubin level (μmol/L)			
6^th^ Month (Mean ± SD)	16.0 ± 10.0	14.3 ± 4.7	0.440
12^th^ Month (Mean ± SD)	16.3 ± 8.9	14.4 ± 3.7	0.329
Normalization of serum Tbil			
6^th^ Month (No. %)	26/31 (%)	22/25 (72.0%)	0.661
12^th^ Month (No. %)	24/31 (89.6%)	22/25 (88.0%)	0.304
ALT (U/L)			
6^th^ Month (Mean ± SD)	38.3 ± 20.6	37.7 ± 15.0	0.903
12^th^ Month (Mean ± SD)	37.2 ± 18.4	34.3 ± 12.2	0.500
Normalization of serum ALT			
6^th^ Month (No. %)	23/31 (74.2%)	21/25 (84.0%)	0.374
12^th^ Month (No. %)	28/31 (90.3%)	20/25 (80.0%)	0.272

After 12^th^ month treatment, the mean serum HBV DNA of the 7 patients who did not achieve undetectable HBV DNA was 4.16 ± 1.16 log10 IU/ml (range 2.77 to 5.71 log10 IU/ml).

### Serologic responses

HBeAg status during treatment was shown in Table 2. The rates of HBeAg loss were similar between the rtA181 group and the rtA181 + rtN236 group (1.3 vs. 5.6%, *P* = 0.448 at the 6^th^ month; 20.8 vs. 11.1%; *P* = 0.403 at the 12th month).

### Biochemical responses

The serum total bilirubin and ALT levels decreased after 6 months ETV rescue therapy in both groups (Table 2). The liver functions and normalization between the two groups did not differ significantly not only at the 6th month but also at the 12th month (*P* > 0.05).

### Responses between different rtA181T/sW172 mutations

The rtA181T substitution was mainly associated with an sW172* substitution, only 15.4% (4/26) had other missense mutations (three with sW172L, one with sW172S).

After emergence of mutant, the serum HBV DNA, HBsAg and ALT levels did not differ significantly between the rtA181T/sW172* mutation group and other missense mutations group (4.16 versus 3.61 log10 IU/mL; *P* = 0.298, 3.52 versus 3.46 log10 IU/mL; *P* = 0.795, 142.5 versus 59.0 U/L; *P* = 0.524, respectively). Throughout the treatment period, serum HBV DNA decreased progressively in the two groups. However, the mean HBV DNA reduction, HBsAg and ALT levels were also similar between the two groups at the 6th month and the 12th month (P > 0.05), as shown in Table 3.

**Table 3:** Responses between different rtA181T/sW172 mutations

Characteristics	rtA181T/sW172* mutation group (*n* = 22)	other missense mutations group (*n* = 4)	*P* value
Before Rescue			
HBV DNA (log_10_ IU/mL)			
Mean ± SD	4.16 ± 2.35	3.61 ± 0.21	0.298
HBsAg (lg IU/ml)			
Mean ± SD	3.52 ± 0.48	3.46 ± 0.40	0.795
ALT (U/L)			
Mean ± SD	142.5 ± 253.8	59.0 ± 40.9	0.524
After Rescue			
HBV DNA Reduction (log_10_ IU/mL)			
6th Month (Mean ± SD)	3.31 ± 2.25	3.61 ± 0.21	0.797
12th Month (Mean ± SD)	3.66 ± 2.18	3.61 ± 0.21	0.918
HBsAg (log_10_ IU/ml)			
6th Month (Mean ± SD)	3.50 ± 0.45	3.47 ± 0.42	0.908
12th Month (Mean ± SD)	3.31 ± 0.50	3.14 ± 0.50	0.529
ALT (U/L)			
6th Month (Mean ± SD)	39.6 ± 19.3	20.5 ± 11.7	0.069
12th Month (Mean ± SD)	39.0 ± 20.0	23.5 ± 3.1	0.142

**Table 4:** Multivariate Analysis of Baseline Factors Predictive of Serum HBV DNA Negativity After 12 months of ETV Rescue Therapy

Varibles	Regression Coefficient	Standard Error	*P* Value	OR (95% CI)
Mutation group	0.487	1.515	0.748	1.627 (0.084–31.666)
HBV DNA level	1.906	0.832	0.022	6.723 (1.315–24.371)
HBsAg level	4.749	3.319	0.152	115.522 (0.173–77176.906)
eAg	−1.696	1.542	0.271	0.183 (0.009–3.765)
Sex	2.256	1.733	0.193	9.545 (0.320–285.099)
Age.	0.064	0.095	0.501	1.066 (0.885–1.284)
Tbil	−0.294	0.183	0.108	0.745 (0.520–1.067)
ALT	0.001	0.016	0.927	1.001 (0.971–1.033)
AST	0.002	0.038	0.951	1.002 (0.931–1.080)

### Baseline predictive factors for serum HBV DNA negativity

After multivariate analysis with adjustment for baseline variables (groups, sex, age, HBsAg level, HBeAg status; serum HBV DNA level, total bilirubin level, ALT and AST level) for all 56 patients, HBV DNA level was the independent covariate to be inversely associated with serum HBV DNA negativity at the 12th month (*P* = 0.022), as shown in Table 4.

## Discussion

In this study, we evaluated the virological, serological, and biochemical outcomes of ETV rescue therapy in patients who developed HBV rtA181T/V mutation strains and compared the ETV rescue therapy efficacy between rtA181T/V mutation and rtA181T/V + rtN236T mutation patients. Serum HBV DNA levels declined to undetectable levels (<50 IU/mL) in 43 of 56 patients (76.8%) after 6 months rescue therapy, and the overall HBV DNA undetectable at the 12th month reached 87.5% (49/56). All therapeutic responses of ETV rescue, including decline of serum HBV DNA, virological, biochemical, and serological responses did not differ significantly between the rtA181T/V group and rtA181T/V + rtN236T group (*P* > 0.05). In addition, HBV DNA level was the predictor of 12 months antiviral outcomes.

The rtA181T/V is a cross-resistance mutation. *in vitro s*tudies found the mutant strain induces a decreased susceptibility to LAM (<10-fold), ADV (2- to 8-fold) and TDF (2- to 3-fold), but it is still susceptible to ETV [10]. We evaluated the efficacy of ETV in rescuing patients with rtA181T/V mutation strain and rtA181T/V + rtN236T mutation strain. In the present study, mean HBV DNA level increased over 4 log_10_ IU/mL after rtA181T/V mutant emergence,but the total bilirubin, ALT and AST level raised moderately, which suggested that the antiviral drug still showed partial effect to rtA181T/V mutant strain, or the mutant virus defective. These explained that many patients who continued previously treatment could partly improve biochemical and virological results after occurring rtA181T/V mutant. This finding was similar to the study in LAM resistance [11].

After ETV rescue therapy, post-treatment HBV DNA level declined significantly. About 87.5% patients achieved undetectable HBV DNA (<50 IU/mL) at the 12th month, which was equivalent to the results of treatment-naïve patients (67% and 90%) [12, 13]. The ALT normalization rate (82.1%) at the 12th month in our analysis was higher than these studies (67 and 78%) [12, 13]. This perhaps resulted from the difference of pre-treatment ALT levels (117U/L vs. 141U/L). In 42 HBeAg positive patients, HBeAg loss after 12 months treatment was 12.5%, approaching to treatment-naïve patients [13]. Buti et al. reported that high pre-treatment baseline HBV DNA levels were risk factor for increased antiviral drug resistance and attenuation of virological response in ADV treatment patients [14]. Our study indicated the similar result that ETV was effective in rtA181T/V mutation patients. HBV DNA level was the only factor that predicted viral clearance.

A recent study has shown that rtA181T mutants with or without S truncation had different virological characteristics. They found the serum levels of HBV DNA and HBsAg in pHBV-rtA181T/sW172* injected mice were significantly lower than that of pHBV-rtA181T/sW172L injected mice [15]. Unfortunately, we could not demonstrate any difference between the two rtA181T mutants. The possible reason was that hepatitis B virus mutant coexisting with wild type virus, which rescues the replication defective in patients [16].

TDF, which is known to be a high barrier HBV inhibitor, has already been used in China. However, Villet has reported rtA181V/T mutants had a slight decrease in susceptibility to TDF [10]. Antiviral efficacy of TDF for HBV rtA181V/T mutants is still unclear in practice [17]. Further investigation of rescue therapy, including TDF, for HBV rtA181V/T mutants alone will be necessary.

In summary, this is the first study to investigate the efficacy of ETV in rescuing CHB patients in rtA181T/V mutation and rtA181T/V + rtN236T mutation patients. The results of the present study indicate that ETV is efficient in rescuing rtA181T/V mutation patients and the HBV DNA level is the only predictor of 12 months antiviral outcomes. There were still some limitations in this study. This research was conducted retrospectively, with an insufficient number of subjects. Thus, there might be a limitation of selection bias. In addition, the follow-up period of our study was relatively short, long-term studies were warranted to evaluate the long-time efficacy of ETV rescue therapy in patients with rtA181T/V mutation.

## Conclusions

Our results indicated that ETV is efficient in rescuing rtA181T/V mutation CHB patients and HBV DNA level could predict viral clearance at the 12th month. That provides clinical evidence for achievement of further antiviral efficacy in NA-related HBV mutants.

## Abbreviations

ADV: Adefovir dipivoxil
ALT: Alanine aminotransferase
AST: Aspartate aminotransferase
CHB: Chronic hepatitis B
ETV: Entecavir
HBeAg: Hepatitis B e antigen
HBsAg: Hepatitis B surface antigen
HBV: Hepatitis B virus
LAM: Lamivudine
LDT: Telbivudine
NAs: Nucleos(t)ide analogs
TDF: Tenofovir
